# Symptoms of Autism, Comorbid Mental Health Conditions and Challenging Behaviors among Toddlers with Down Syndrome at Low Risk for ASD—Characterization Using the BISCUIT—Parts 1–3

**DOI:** 10.3390/ijerph182010684

**Published:** 2021-10-12

**Authors:** Ewa Pisula, Alicja Niedźwiecka

**Affiliations:** Department of Health and Rehabilitation Psychology, Faculty of Psychology, University of Warsaw, 00-183 Warsaw, Poland; ewa.pisula@psych.uw.edu.pl

**Keywords:** Down syndrome, developmental disorders, autism spectrum disorders, BISCUIT, Q-CHAT, mental health, challenging behaviors

## Abstract

*Background*: Autism spectrum disorder (ASD) may coexist with Down syndrome (DS). Most studies on this topic involve school-age children, adolescents, or adults with DS. This study looked at ASD symptoms, other mental health problems, and challenging behaviors in toddlers with DS at low risk of ASD. *Methods*: We used screening tools for autism in toddlers; BISCUIT–Parts 1–3 and Q-CHAT. We compared four groups of children aged 17–37 months: DS, ASD, Atypical Development (AD), and Typically Developing (TD). *Results*: Children with DS showed lower symptoms of ASD than children with ASD (without DS) and higher than TD children, except for repetitive behaviors/restricted interests. For comorbid mental health problems and difficult behaviors, children with DS scored lower than children with ASD. There were no differences between children with DS and TD children in this regard. *Conclusions*: The study results indicate that BISCUIT–Parts 1–3 are valid instruments to differentiate toddlers with DS from toddlers with ASD. However, they also show that toddlers with DS at low ASD risk are a very heterogeneous group when the ASD symptoms are considered. Autistic characteristics should be taken into account in supporting young children with this genetic condition.

## 1. Introduction

Autism spectrum disorder (ASD) is a complex neurodevelopmental disorder characterized by early-onset socio-communication difficulties and restricted patterns of behavior, activity, and interests [1,2]. Children with ASD experience difficulties in building age-appropriate social relationships and participating in reciprocal social interactions [1]. They show limited abilities to recognize and interpret social cues and respond to them appropriately [3]. Less effectively than typically developing peers, they read facial expressions, body language, and other non-verbal signals crucial for communication with others. These difficulties are accompanied by limited, repetitive, and schematic ways of using objects, narrow, intense interests, and usually also atypical sensory sensitivity [4]. ASD is now recognized as one of the most common developmental problems in children. Its frequency is estimated at 1–2% of the population [5,6].

Autism is also defined as a behavioral syndrome that occurs relatively frequently in certain genetic syndromes [7] (for a review, see [8]). Among these disorders is Down syndrome (DS), the most common cause of which is a trisomy of chromosome 21 [9].

DS occurs in approximately 1 of 800 births and results in severe medical conditions (e.g., heart disease, immunodeficiency, hearing deficits) and neurodevelopmental problems (e.g., intellectual disability, decreased social awareness, decreased motor coordination) [10]. DS has a distinct phenotype that involves delays and deficits in various areas of development, such as cognition (average IQ of 50) and speech, motor, and social development, with learning delays accelerating at ages 2–4 years [11]. Specifically, the cognitive phenotype associated with DS is characterized by relatively low processing speed, difficulties with syntax and morphosyntax, and weakness of short-term verbal memory relative to visuospatial memory [12]. Concurrently, considerable individual differences can be observed, including IQ scores and language [13].

Studies among children and adolescents with DS revealed a prevalence of ASD in 6–7% of the sample [14,15]. However, a systematic review and meta-analysis by Richards et al. [8] showed a 16% prevalence of ASD in the DS population. In addition, more recent studies have shown that as many as 38–42% [16,17], respectively, of people with DS meet at least some diagnostic criteria for ASD.

The diagnosis of ASD in children with Down syndrome can be problematic since socialization and communication problems present in some children with DS may be associated with IQ deficits and sensory impairments [18,19]. Consequently, collecting information about ASD symptoms in the population of children with DS is still useful. Research focuses primarily on investigating the differences between children with dual ASD + DS diagnosis and children with only ASD or DS. In one of these studies, the Autism Diagnostic Observation Schedule–2 [20] was used to assess the symptoms of ASD among children and adolescents with DS aged 5–17 years [21]. A considerable number of participants, 15 out of 41, met the diagnostic criteria for ASD. Participants with DS + ASD had more elevated symptoms in all three core domains of ASD (verbal and non-verbal communication, reciprocal social interaction, stereotyped behaviors, and restricted interests) compared to participants with DS only. The two items that best differentiated between the groups with and without ASD were the frequency of vocalization directed to others and the unusual eye contact.

Several more studies examined the symptoms of ASD among children with DS and ASD, e.g., [22,23,24,25,26]. However, little attention has been given to the symptoms of ASD and co-occurring problems among young children with DS. This paper focuses on symptoms of ASD among toddlers with DS at low risk for ASD (without diagnosed or suspected ASD, and without ASD diagnosis in their closest relatives). Autistic-like symptomatology in people with DS at low risk for ASD was studied by Channell and colleagues [27], using the Social Responsiveness Scale (SRS) [28]. They found that adolescents with DS (aged 10–21) scored significantly higher than a normative sample or just below the cut-off score. Individuals with DS had relatively low symptoms in the areas of social awareness and motivation and the most elevated symptoms in the areas of autistic mannerisms and social cognition. Social communication scores were somewhere in between. In another study by Channell [29], school-aged children (aged 6–11) with DS at low risk for ASD had significantly elevated autistic-like symptoms compared to the normative population sample. This effect was present in all of the SRS-2 [30] subdomains. In general, the results were similar to the previous study by Channell et al. [27]. Still, the author notes that some subtle differences occurred and might indicate developmental differences across different ages of children with DS. These differences concerned, among others, social awareness, for which 49% in the school-aged children scored within the elevated symptomatology range, compared to only 28% in the adolescent group.

Autistic symptoms in toddlers with DS were studied by Hepburn, Philofsky, Fidler, and Rogers [31] using the Autism Diagnostic Observation Schedule-Generic (ADOS-G) [32] and the Autism Diagnostic Interview—Revised (ADI-R) [33]. Most of the children were examined twice, at 2 and 4 years of age. Five out of 20 subjects at both time points met the diagnostic criteria for autism spectrum disorders or autistic disorder, and several others exhibited difficulties in communication and play typical of autism but had no problems in core social relatedness.

Interesting information on ASD symptoms in toddlers with DS can be obtained from the Baby and Infant Screen for Children with Autism Traits Battery (BISCUIT) [34]. This screening tool, intended for supporting the diagnostic process in young children, allows for measuring the severity of ASD symptoms and symptoms of other comorbid mental health conditions and challenging behaviors often found in children with ASD. Comorbid mental health conditions, including attention-deficit/hyperactivity disorder, oppositional-defiant, and conduct disorders, also occur in some children with DS [16,35]. Behavioral problems can be observed in this population in various areas of functioning: feeding, sleep, toilet training, and socialization [36,37]. An assessment with the Child Behavior Checklist [38] revealed that some children and adolescents with DS aged 4–19 years had social attention problems and thought problems [39]. Stereotypies are frequent in DS, see a review in [40]; however, they are less severe than in ASD [19]. At the same time, children with DS show fewer maladaptive behaviors and suffer from comorbid psychopathologies less frequently than children with intellectual disabilities without DS [36]. Cappone with colleagues [41,42] noted several differences in behavioral problems between children DS + ASD and children DS + stereotypic movement disorder or DS + disruptive behaviors. However, the age of the respondents in that study was in a wide range of 2–24 years. A better understanding of the clinical picture of ASD symptoms and comorbid problems in toddlers with DS could improve the early diagnosis of developmental difficulties in this group of children and help support those children with DS who experience difficulties typical of ASD.

### Current Study

In this study, we examined the symptoms of ASD among toddlers with DS as compared with toddlers with ASD (the ASD group), atypical development (AD group), and typically developing controls (TD group) using the BISCUIT-Part 1 [34]. We concentrated on children with DS at low risk for ASD. Furthermore, we investigated the occurrence of comorbid mental health conditions (attention-deficit/hyperactivity disorder, tic disorder, obsessive-compulsive disorder, specific phobia, and eating/feeding difficulties) with the BISCUIT-Part 2 and challenging behaviors (aggression, disruption, self-injury, and stereotypies) using the BISCUIT-Part 3. We aimed to determine differences between children with DS and those with ASD or TD. Additionally, we used a screening tool for ASD, the Quantitative Checklist for Autism in Toddlers, Q-CHAT [43], to examine whether the BISCUIT-Part 1 scores were associated with the most commonly observed symptoms of ASD among toddlers.

With respect to the BISCUIT-Parts 1–3 scores, we expected the children with DS to score lower than those with ASD and higher than TD controls. We made no specific predictions regarding the differences between the DS and AD groups. We expected those scores to be significantly and positively correlated with the Q-CHAT scores. We also checked associations with age to better understand the symptoms assessed by BISCUIT-Parts 1–3 among toddlers with DS.

## 2. Materials and Methods

### 2.1. Design

Questionnaires were completed by the primary caregiver (85% mothers) as part of several studies at home or in daycare, early intervention centers, and clinics across Poland. The number of filled in BISCUIT-Parts 1–3, Q-CHAT, and socio-demographic forms varied. For this reason, sample sizes will be reported for each analysis.

Participants were recruited by professionals providing daycare or early diagnosis and intervention through flyers and posters distributed in health care centers, nurseries, and through media ads. All parents gave written informed consent. The studies were approved by the local institution’s ethics committee and conformed to the Declaration of Helsinki.

### 2.2. Participants and Procedure

There were 578 participants in the entire sample. Table 1 presents some basic socio-demographic characteristics of the sample.

The mean age of participants was 26.45 months (*SD* = 5.312, min = 17, max = 37). There were some significant age differences between groups, *F* = (3, 574) = 5.755, *p* < 0.001, ŋ_p_^2^ = 0.029. Bonferroni-corrected pairwise comparisons revealed that mean age was higher in the ASD group than in the TD group, *t* = 1.981, *p* = 0.001, BCa 95% CI [0.693, 3.323]. There were no other significant age differences between the groups, *p* > 0.05.

Boys outnumbered girls in the ASD group, *Chi*^2^(1, 220) = 61.164, *p* < 0.001, and in the AD group, *Chi*^2^(1, 93) = 4.742, *p* = 0.029 (male to female ratio: 3.231and 1.583, respectively). There were no significant differences in the number of boys and girls in the DS group or the TD group, *p* > 0.05 (male to female ratio: 1.435 and 1.069, respectively).

All children with DS (N = 56) had received a medical diagnosis based on genetic testing and were under the care of early intervention centers. None of the children had biological siblings with ASD (one child had a distant cousin diagnosed with ASD and one had a step sibling with the same diagnosis). Parents were contacted through early intervention centers.

The ASD group (N = 220) included children with clinical diagnosis of childhood autism or pervasive developmental disorder, unspecified. The diagnosis was given by child psychiatrists based on ICD-10 criteria [44]. Children aged <20 months (*n* = 26) currently undergoing the diagnostic process were also included in the sample. They were referred with suspected ASD to diagnostic centers by paediatricians, with initial findings suggesting a high probability of a final diagnosis of ASD. Parents were contacted through diagnostic centers specializing in ASD and early intervention centers.

The atypical development group (N = 93) consisted of children referred to diagnostic centers mostly due to significantly delayed speech and impaired motor development (e.g., cerebral palsy), as well as central nervous system defects (e.g., agenesis of corpus callosum). The group was highly heterogeneous in terms of the type of medical problems and psychological development deficits. Nine children had elevated familial risk for ASD (siblings with ASD diagnosis). Parents were contacted through early intervention centers.

The typically developing group (N = 209) was recruited through nurseries and preschools. The inclusion criteria for this group were age (17–37 months), no diagnosed developmental disorders and no developmental difficulties reported by parents or educators. Fifteen children in the group had increased familial risk for ASD (six brothers and three sisters diagnosed with ASD).

### 2.3. Instruments

The Baby and Infant Screen for Children with Autism Traits—Parts 1–3 (BISCUIT–Parts 1–3) [34], the Quantitative Checklist for Autism in Toddlers [43] and a demographic questionnaire were used in the study in the paper-and-pencil form.

The BISCUIT is a three-part set of questionnaires for the assessment of children aged 17–37 months. All three parts have a high internal consistency [45,46]. The BISCUIT–Part 1 contains 62 items and assesses the symptoms of autism spectrum disorders, and has high validity, sensitivity, and specificity in identifying ASD [34]. In the BISCUIT–Part 1, informants rate their child’s behavior compared to same-aged peers on a Likert scale ranging from 0 to 2 (0—not different; no impairment, 1—somewhat different; mild impairment, 2—very different; severe impairment). The total score illustrating the intensity of the ASD symptoms is calculated. Moreover, in the factor analysis, three distinct factors were identified: socialization/nonverbal communication, repetitive behavior/restricted interests, and verbal communication. They form three subscales of high reliability [47].

The BISCUIT–Part 2 contains 57 items and assesses the symptoms of comorbid mental health conditions (attention-deficit/hyperactivity disorder, tic disorder, obsessive-compulsive disorder, specific phobia, and eating/feeding difficulties; [48]. The child’s behavior is rated on a 3-point Likert scale from 0 to 3 (0—not a problem or impairment; not at all, 1—mild problem or impairment, 2—severe problem or impairment). The total score may be obtained, and the scores in the following subscales: Tantrum/conduct behavior, Inattention/Impulsivity, Avoidance Behavior, Anxiety/Repetitive Behavior, and Eating/Sleep Problems.

The BISCUIT–Part 3 contains 15 items and assesses challenging behaviors (aggression, disruption, self-injury, and stereotypies [49]. The child’s behavior is rated on a 3-points Likert scale (the same as in the BISCUIT–Part 2). The total score is calculated, and the scores in three subscales may also be obtained: aggressive/disruptive behaviors, stereotypic behaviors, and self-injurious behaviors.

The Polish language versions of the BISCUIT–Parts 1–3 were developed using a back-translation procedure with the authorization of the original developers [50]. The translation process was a part of the international research project on early symptoms of ASD [48]. In this study, the alpha coefficient for the BISCUIT–Part 1 total score was 0.985 for the entire sample, BISCUIT–Part 2 total score 0.835, and BISCUIT–Part 3 total score of 0.846. In the BISCUIT–Part 1, Cronbach’s Alpha was 0.98 for socialization/non-verbal communication and 0.95 for both repetitive behavior/restricted interests and verbal communication. In the BISCUIT–Part 2 the reliability coefficients were high or at least satisfactory: for tantrum/conduct behavior α = 0.89, inattention/impulsivity 0.92, avoidance behavior 0.88, anxiety/repetitive behavior 0.87, and eating/sleep problems 0.76. In BISCUIT–Part 3, the alpha coefficients were satisfactory for two subscales: aggressive/disruptive behaviors and stereotypic behaviors (0.80 and 0.77, respectively) and low for self-injurious behaviors (0.50). The authors of BISCUIT reported a very similar coefficient level for the original BISCUIT subscales, including low reliability of the self-injurious behaviors [34]. This subscale has two items only, which may explain the low value of the alpha coefficient.

The Q-CHAT is a 25-item screening tool for ASD to be completed by caregivers of toddlers. Items are scored on a Likert-like scale, with higher scores indicating more severe symptoms. The original version of the instrument has high reliability [43]. The Polish language version was developed with the authors’ consent, using the back translation procedure. In this study, the Cronbach’s alpha coefficient for Q-CHAT total score was 0.826.

The demographic questionnaire included questions regarding children’s age, gender, gestational age at birth, number of siblings, developmental concerns and health issues, parents’ education level, and the family’s place of residence.

### 2.4. Analyses

First, we examined the distribution of BISCUIT–Parts 1–3 and Q-CHAT scores for the entire sample. Next, we ran zero-order correlations between participants’ age and BISCUIT–Parts 1–3 and Q-CHAT scores. Finally, we run GLM analyses with age as a covariate to compare BISCUIT–Parts 1–3 and Q-CHAT scores among the four groups of participants (DS, ASD, AD, TD).

## 3. Results

### 3.1. Distributions of BISCUIT and Q-CHAT Scores

**Figure 1:**
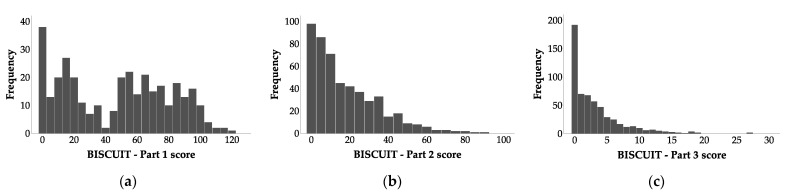
Distributions of: (**a**) BISCUIT–Part 1 scores; (**b**) BISCUIT–Part 2 scores; (**c**) BISCUIT–Part 3 scores for the entire sample.

Figure 1 presents the distribution of BISCUIT–Part 1–3 scores. In case of BISCUIT–Part 1 (Figure 1a; *n* = 341, *M* = 47.173, *SD* = 33.269) the distribution significantly differed from the normal distribution, *D* = 0.127, *p* < 0.001, skewness 0.075 (SE = 0.132), kurtosis −1.302 (SE = 0.263). For BISCUIT–Part 2 scores (Figure 1b, *n* = 510, *M* = 18.128, *SD* = 17.868) the distribution also significantly differed from the normal distribution, *D* = 0.155, *p* < 0.001, skewness 1.349 (SE = 0.108), kurtosis of 1.969 (SE = 0.216). The same was in case of BISCUIT–Part 2 (Figure 1c, *n* = 576, *M* = 3.162, *SD* = 4.053). The distribution significantly differed from the normal distribution, *D* = 0.218, *p* < 0.001, skewness 2.086 (SE = 0.102), kurtosis of 5.799 (SE = 0.203).

**Figure 2:**
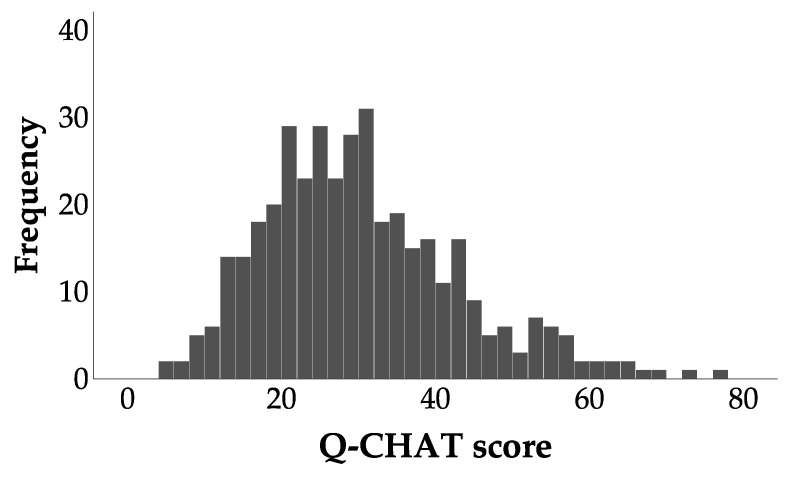
Distribution of Q-CHAT scores.

Figure 2 presents the distribution of Q-CHAT scores for the entire sample (*n* = 393, *M* = 30.000, *SD* = 12.960). The distribution resembled but significantly differed from the normal distribution, *D* = 0.090, *p* < 0.001, skewness 0.808 (SE = 0.123), kurtosis −0.767 (SE = 0.246).

### 3.2. Group Comparisons

**Figure 3:**
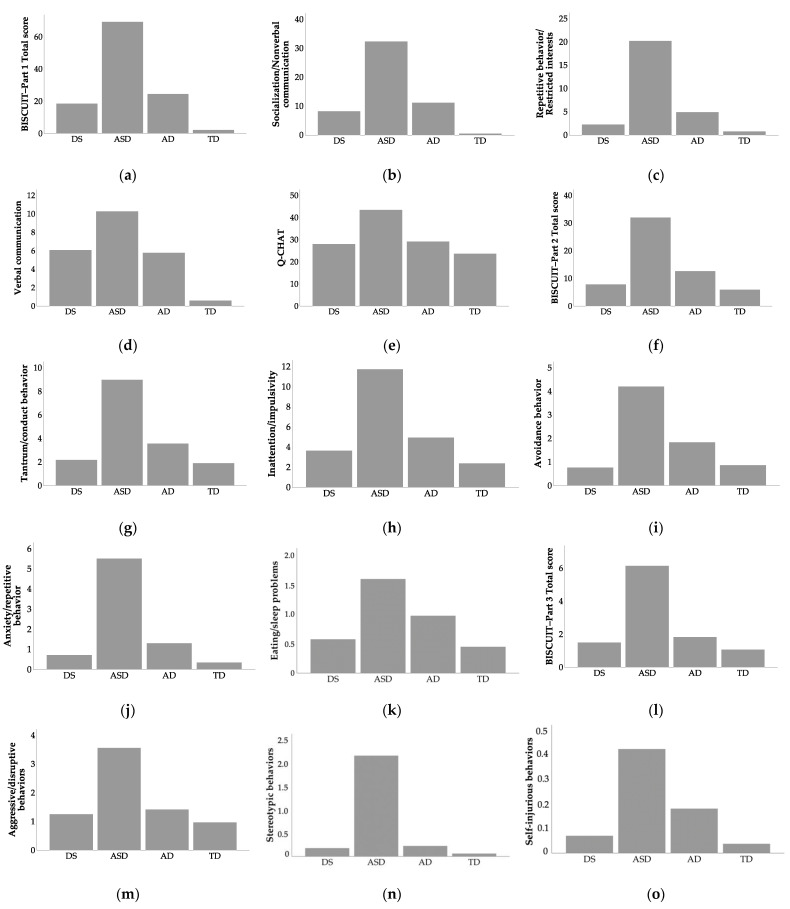
Mean scores of the 4 group of participants in: (**a**) BISCUIT–Part 1; (**b**) BISCUIT Part–1 Socialization/Nonverbal communication; (**c**) BISCUIT Part–1Repetitive behavior/Restricted interests; (**d**) BISCUIT Part–1 Verbal communication; (**e**) Q-CHAT; (**f**) BISCUIT–Part 2; (**g**) BISCUIT–Part 2 Tantrum/conduct behavior; (**h**) BISCUIT–Part 2 Inattention/impulsivity; (**i**) BISCUIT–Part 2 Avoidance behavior; (**j**) BISCUIT–Part 2 Anxiety/repetitive behavior; (**k**) BISCUIT–Part 2 Eating/sleep problems; (**l**) BISCUIT–Part 3 Total score; (**m**) BISCUIT–Part 3 Aggressive/disruptive behaviors; (**n**) BISCUIT–Part 3 Stereotypic behaviors; (**o**) BISCUIT–Part 3 Self-injurious behaviors. Higher scores indicate more elevated symptoms. Abbreviations: DS, Down syndrome; ASD, autism spectrum disorders; AD, atypical development; TD, typical development.

Figure 3 illustrates mean scores of the DS, ASD, AD, and TD groups in BISCUIT and Q-CHAT scores. Table A1 in Appendix A includes descriptive statistics for BISCUIT and Q-CHAT scores.

### 3.3. Symptoms of Autism Spectrum Disorders

#### 3.3.1. BISCUIT–Part 1

BISCUIT–Part 1 scores were available for 341 participants (DS *n* = 54, ASD *n* = 203, AD *n* = 37, TD *n* = 47).

A GLM a nalysis revealed a significant main effect of group for the BISCUIT–Part 1 total score, *F*(3, 336) = 244.470, *p* < 0.001, ŋ_p_^2^ = 0.686. There was no significant main effect of age, *p* > 0.05. Planned comparisons revealed that toddlers in the DS group scored significantly higher than those in the TD group, *t* = 16.389, *p* < 0.001, BCa 95% CI [9.028, 23.751]. They scored significantly lower than toddlers in the ASD group, *t* = −50.788, *p* < 0.001, BCa 95% CI [−56.438, −45.138], and those in the AD group, *t* = −5.947, *p* < 0.001, BCa 95% CI [−13.832, 1.937]. Toddlers in the ASD group scored significantly higher than toddlers in the AD, *t* = 44.841, *p* < 0.001, BCa 95% CI [38.228, 51.454], and TD groups, *t* = 67.178, *p* < 0.001, BCa 95% CI [61.205, 73.150]. Toddlers in the AD group scored higher than those in the TD group, *t* = 22.337, *p* < 0.001, BCa 95% CI [14.212, 30.462].

The main group effects also occurred in the BISCUIT–Part 1 subscales. In the case of socialization/nonverbal communication, [*F*(3, 336) = 228.785, *p* < 0.001, ŋ_p_^2^ = 0.666], toddlers with DS scored lower than ASD group, *t* = −24.138, *p* < 0.001, and higher that TD group, *t* = 7722, *p* < 0.001. In the repetitive behavior/restricted interests subscale, *F*(3, 336) = 194.246, *p* < 0.001, ŋ_p_^2^ = 0.629, toddlers with DS differed only from children with ASD (*t* = 17.962, *p* < 0.001). In the verbal communication subscale, *F*(3, 336) = 147.384, *p* < 0.001, ŋ_p_^2^ = 0.562, toddlers with DS scored lower than toddlers with ASD (*t* = −4.2, *p* < 0.001) and higher than TD group (*t* = 5.47, *p* < 0.001).There were no significant differences between DS and AD groups in any of the subscales.

#### 3.3.2. Q-CHAT

Q-CHAT scores were available for 393 participants (DS *n* = 42, ASD *n* = 74, AD *n* = 96, TD *n* = 181). A GLM analysis revealed a significant main effect of group for the Q-CHAT scores, *F*(3, 388) = 84.799, *p* < 0.001, ŋ_p_^2^ = 0.396. There was also a significant, albeit very weak, main effect of age, *F*(1, 388) = 16.285, *p* < 0.001, ŋ_p_^2^ = 0.040.

Planned comparisons revealed that toddlers in the DS group scored significantly higher than those in the TD group, *t* = 5.517, *p* = 0.002, BCa 95% CI [2.089, 8.945]. They scored significantly lower than toddlers in the ASD group, *t* = −14.683, *p* < 0.001, BCa 95% CI [−18.358, −11.009]. There was no significant difference between the scores of toddlers in the DS and AD groups, *p* > 0.05. Toddlers in the ASD group scored significantly higher than toddlers in the AD, *t* = 14.633, *p* < 0.001, BCa 95% CI [11.574, 17.692], and TD groups, *t* = 20.201, *p* < 0.001, BCa 95% CI [17.701, 22.700]. Toddlers in the AD group scored significantly higher than those in the TD group, *t* = 5.568, *p* < 0.001, BCa 95% CI [2.842, 8.293].

### 3.4. Symptoms of Comorbid Mental Health Conditions

BISCUIT–Part 2 scores were available for 510 participants (DS *n* = 54, ASD *n* = 202, AD *n* = 76, TD *n* = 178).

#### 3.4.1. BISCUIT—Part 2 Total Score

A GLM analysis revealed a significant main effect of group for the BISCUIT–Part 2 total score, *F*(3, 505) = 135.774, *p* < 0.001, ŋ_p_^2^ = 0.446. There was also a significant, albeit very weak, main effect of age, *F*(1, 505) = 4.162, *p* = 0.042, ŋ_p_^2^ = 0.008.

Planned comparisons did not reveal any significant differences between toddlers in the DS group and those in the TD group, *p >* 0.05. Toddlers in the DS group scored significantly lower than toddlers in the ASD group, *t* = −24.114, *p* < 0.001, BCa 95% CI [−28.080, −20.147]. They also scored significantly lower than the AD group, *t* = −5.107, *p* = 0.030, BCa 95% CI [−9.720, −0.494]. Toddlers in the ASD group scored significantly higher than toddlers in the AD group, *t* = 19.006, *p* < 0.001, BCa 95% CI [15.510, 22.503], and TD groups, *t* = 25.886, *p* < 0.001, BCa 95% CI [23.194, 28.578]. Toddlers in the AD group scored significantly higher than those in the TD group, *t* = 6.879, *p* < 0.001, BCa 95% CI [3.330, 10.428].

#### 3.4.2. Tantrum/Conduct Behavior

A GLM analysis revealed a significant main effect of group for the BISCUIT–Part 2 Tantrum/conduct behavior scores, *F*(3, 505) = 70.973, *p* < 0.001, ŋ_p_^2^ = 0.297. There was also a significant, albeit very weak, main effect of age, *F*(1, 505) = 5.483, *p* = 0.020, ŋ_p_^2^ = 0.011.

Planned comparisons did not reveal any significant differences between toddlers in the DS group and those in the TD group, *p >* 0.05. Toddlers in the DS group scored significantly lower than toddlers in the ASD group, *t* = −6.940, *p* < 0.001, BCa 95% CI [−8.355, −5.535]. The DS group also scored lower than the AD group, *t* = −2.213, *p* = 0.008, BCa 95% CI [−3.858, −0.568]. Toddlers in the ASD group scored significantly higher than toddlers in the AD group, *t* = 4.727, *p* < 0.001, BCa 95% CI [3.480, 5.974], and TD groups, *t* = 6.476, *p* < 0.001, BCa 95% CI [5.516, 7.436]. Toddlers in the AD group scored higher than those in the TD group, *t* = 1.748, *p* = 0.007, BCa 95% CI [0.482, 3.014].

#### 3.4.3. Inattention/Impulsivity

A GLM analysis revealed a significant main effect of group for the BISCUIT–Part 2 Inattention/Impulsivity scores, *F*(3, 505) = 123.920, *p* < 0.001, ŋ_p_^2^ = 0.424. There was no significant main effect of age, *p* > 0.05.

Planned comparisons did not show any significant differences between toddlers in the DS group and those in the TD group. Toddlers in the DS group scored lower than those in the ASD group, *t* = −8.175, *p* < 0.001, BCa 95% CI [−9.697, −6.652]. There was no significant difference between the DS and AD groups, *p* > 0.05. Toddlers in the ASD group scored significantly higher than toddlers in the AD group, *t* = 6.893, *p* < 0.001, BCa 95% CI [5.552, 8.235], and TD groups, 9.662, *p* < 0.001, BCa 95% CI [8.629, 10.695]. Toddlers in the AD group scored significantly higher than toddlers in the TD group, *t* = 2.768, *p* < 0.001, BCa 95% CI [1.406, 4.130].

#### 3.4.4. Avoidance Behavior

A GLM analysis showed a significant main effect of group for the BISCUIT–Part 2 Avoidance behavior scores, *F*(3, 505) = 120.102, *p* < 0.001, ŋ_p_^2^ = 0.416. There was a significant, albeit small, effect of age, *F* = [1, 505] = 6.508, *p* = 0.011, ŋ_p_^2^ = 0.013.

Planned comparisons did not reveal any significant differences between toddlers in the DS group and those in the TD group, *p* > 0.05. Toddlers in the DS group scored lower than those in the ASD group, *t* = −4.673, *p* < 0.001, BCa 95% CI [−3.860, −4920]. There was no significant difference between the DS and AD groups. Toddlers in the ASD group scored significantly higher than toddlers in the AD group, *t* = 4.000, *p* < 0.001, BCa 95% CI [3.283, 4.717], and TD groups, *t* = 4.920, *p* < 0.001, BCa 95% CI [4.368, 5.472]. Toddlers in the AD group scored significantly higher than those in the TD group, *t* = 0.920, *p* = 0.013, BCa 95% CI [0.192, 1.648].

#### 3.4.5. Anxiety/Repetitive Behavior

A GLM analysis revealed a significant main effect of group for the BISCUIT–Part 2 Inattention/Impulsivity scores, *F*(3, 505) = 42.877, *p* < 0.001, ŋ_p_^2^ = 0.203. There was no significant main effect of age.

Planned comparisons did not reveal any significant differences between toddlers in the DS group and those in the TD group, *p* > 0.05. Toddlers in the DS group scored lower than those in the ASD group, *t* = −3.384, *p* < 0.001, BCa 95% CI [−4.283, −2.486]. There was no significant difference between the DS and AD groups. Toddlers in the ASD group scored significantly higher than toddlers in the AD group, *t* = 2.392, *p* < 0.001, BCa 95% CI [1.600, 3.183], and TD groups, *t* = 3.200, *p* < 0.001, BCa 95% CI [2.590, 3.809]. There was no significant difference between the AD and TD groups, *p* > 0.05.

#### 3.4.6. Eating/Sleep Problems

A GLM analysis indicated a significant main effect of group for the BISCUIT—Part 2 Eating/sleep problems, *F*(3, 505) = 18.214, *p* < 0.001, ŋ_p_^2^ = 0.098. There was no significant main effect of age, *p* > 0.05

Planned comparisons did not show any significant differences between toddlers in the DS group and those in the TD group, *p* > 0.05. Toddlers in the DS group scored lower than those in the ASD group, *t* = −0.979, *p* < 0.001, BCa 95% CI [−1.441, −0.517]. There was no significant difference between the DS and AD groups, *p* > 0.05. Toddlers in the ASD group scored significantly higher than toddlers in the AD group, *t* = 0.772, *p* = 0.001, BCa 95% CI [0.315, 1.129], and TD groups, *t* = 1.128, *p* < 0.001, BCa 95% CI [0.814, 1.441]. There was no significant difference between the AD and TD groups.

### 3.5. Symptoms of Challenging Behaviors

BISCUIT–Part 3 scores were available for 576 participants (DS *n* = 56, ASD *n* = 219, AD *n* = 93, TD *n* = 208).

#### 3.5.1. BISCUIT–Part 3 Total Score

A GLM analysis revealed a significant main effect of group for the BISCUIT–Part 3 total score, *F*(3, 571) = 91.148, *p* < 0.001, ŋ_p_^2^ = 0.324. There was also a significant, albeit very weak, main effect of age, *F*(1, 571) = 4.305, *p* = 0.038, ŋ_p_^2^ = 0.007.

Planned comparisons did not reveal any significant differences between toddlers in the DS group and those in the TD group, *p >* 0.05. Toddlers in the DS group scored significantly lower than toddlers in the ASD group, *t* = −4.618, *p* < 0.001, BCa 95% CI [−5.590, −3.646]. There was no significant difference between the DS and AD groups, *p* > 0.05. Toddlers in the ASD group scored significantly higher than toddlers in the AD group, *t* = 4.223, *p* < 0.001, BCa 95% CI [3.416, 5.030], and TD groups, *t* = 4.959, *p* < 0.001, BCa 95% CI [4.323, 5.595]. There was no significant difference between the AD and TD groups, *p* > 0.05.

#### 3.5.2. Aggressive/Disruptive Behaviors

A GLM analysis showed a significant main effect of group for the BISCUIT—Part 3 Aggressive/disruptive behaviors, *F*(3, 571) = 38.706, *p* < 0.001, ŋ_p_^2^ = 0.169. There was also a significant, albeit very weak, main effect of age, *F*(1, 571) = 5.914, *p* = 0.015, ŋ_p_^2^ = 0.010.

Planned comparisons did not reveal any significant differences between toddlers in the DS group and those in the TD group, *p >* 0.05. Toddlers in the DS group scored significantly lower than toddlers in the ASD group, *t* = −2.294, *p* < 0.001, BCa 95% CI [−3.040, −1.549]. There was no significant difference between the DS and AD groups, *p* > 0.05. Toddlers in the ASD group scored significantly higher than toddlers in the AD group, *t* = 2.065, *p* < 0.001, BCa 95% CI [1.446, 2.684], and TD groups, *t* = 2.495, *p* < 0.001, BCa 95% CI [2.007, 2.983]. There was no significant difference between the AD and TD groups, *p* > 0.05.

#### 3.5.3. Stereotypic Behaviors

A GLM analysis revealed a significant main effect of group for the BISCUIT–Part 3 Stereotypic behavior scores, *F*(3, 571) = 165.588, *p* < 0.001, ŋ_p_^2^ = 0.465. There was no significant effect of age.

Planned comparisons did not indicate any significant differences between toddlers in the DS group and those in the TD group, *p* > 0.05. Toddlers in the DS group scored significantly lower than toddlers in the ASD group, *t* = −1.970, *p* < 0.001, BCa 95% CI [−2.278, −1.663]. There was no significant difference between the DS and AD groups, *p* > 0.05. Toddlers in the ASD group scored significantly higher than toddlers in the AD group, *t* = 1.917, *p* < 0.001, BCa 95% CI [1.662, 2.173], and TD groups, *t* = 2.078, *p* < 0.001, BCa 95% CI [1.877, 2.280]. There was no significant difference between the AD and TD groups, *p* > 0.05.

#### 3.5.4. Self-Injurious Behaviors

A GLM analysis revealed a significant main effect of group for the BISCUIT—Part 3 self-injurious behavior scores, *F*(3, 571) = 20.017, *p* < 0.001, ŋ_p_^2^ = 0.095. There was no significant effect of age.

Planned comparisons did not show any significant differences between toddlers in the DS group and those in the TD group, *p* > 0.05. Toddlers in the DS group scored significantly lower than toddlers in the ASD group, *t* = −0.353, *p* < 0.001, BCa 95% CI [−0.509, −0.197]. There was no significant difference between the DS and AD groups, *p* > 0.05. Toddlers in the ASD group scored significantly higher than toddlers in the AD group, *t* = 0.241, *p* < 0.001, BCa 95% CI [0.111, 0.371], and TD groups, *t* = 0.385, *p* < 0.001, BCa 95% CI [0.283, 0.487]. The AD group scored higher than the TD group, *t* = 0.144, *p* = 0.030, BCa 95% CI [0.014, 0.274].

### 3.6. Correlations between Participants’ Age, BISCUIT–Parts 1–3 and Q-CHAT Scores

Table 2 presents a matrix of zero-order correlations between participants, age, and BISCUIT–Parts 1–3 and Q-CHAT scores. Age was correlated with some BISCUIT and Q-CHAT scores, but the correlations were very weak. The correlation between BISCUIT–Part 1 total score and Q-CHAT was strong (r = 0.788). Mostly the correlations between BISCUIT total scores and subscale scores were moderate to strong.

## 4. Discussion

This study focused on children with DS aged 17–37 months at low risk for ASD, aiming to characterize them in the context of ASD symptoms and problems often coexisting with ASD. We examined ASD symptoms, comorbid mental health conditions, and behavioral problems, comparing toddlers with DS with peers with ASD, atypical and typical development. We used BISCUIT–Parts 1–3 and Q-CHAT, tools useful in the screening for ASD.

The DS group’s ASD symptom severity was consistent with expectations. Overall, children with DS showed less severe symptoms of ASD than children with ASD diagnosis, but more elevated symptoms than their TD peers. This was true both of the overall severity of ASD symptoms (total score), and individual subscales in BISCUIT–Part 1. Effect size analysis of between-group differences showed large differences between the ASD and the other groups (ŋ_p_^2^ from 0.6 to 0.7). Thus, our findings support the validity of BISCUIT–Part 1 in measuring the symptoms of ASD in toddlers. In the analyses of psychometric properties of BISCUIT–Part 1 to date, children with DS were included in the atypical development group, e.g., [48,49], of which they only made up a small percentage. The analyses presented here offer a more comprehensive insight into the differences in BISCUIT–Part 1 scores between children with DS and children with ASD. We cannot comment on BISCUIT cutoff scores, as they have yet to be calculated for the Polish population. Matson with colleagues [51] report that the best specificity and sensitivity in terms of differentiating between pervasive developmental disorder not otherwise specified and TD in the US was determined to be the score of 22. In our study, 12 children with DS scored 22 or more. To a certain degree, this reflects the extent of clinical severity of ASD symptoms in the group of children with DS, although direct application of cut-off values obtained in another country, and only with respect to the pervasive developmental disorder not otherwise specified, is unwarranted. It is worth pointing out, however, the high variance in the severity of ASD symptoms in children with DS in our study. The range for BISCUIT–Part 1 total score in this group was from 7 to 67 points (total range 0–124 points). Symptom severity was unrelated to age. Unfortunately, we have not measured mental age and thus we were unable to assess its effects.

New information was also obtained from other between-group comparisons in BISCUIT–Part 1 subscales. The DS group scored higher than TD group in socialization/non-verbal communication, and verbal communication subscales; no differences between these groups were observed in repetitive behavior/restricted interests subscale. Thus, symptoms of one of the domains key to ASD diagnosis, namely repetitive, restricted patterns of behavior, rated by parents, were as common in toddlers with DS as in their typically developing peers. This subscale’s items include, among others, abnormal fascination with the movement of spinning objects or abnormal preoccupation with parts of objects, as well as use of facial expressions, isolates self, eye to eye gaze or maintaining eye contact. Other studies show the large number of restricted, repetitive patterns of behavior in individuals with DS [52]. However, in a study of school-aged children and adolescents with DS and typically developing children matched for mental age, Evans and Gray [53] found similar numbers of routines in both groups. The authors believe these results may suggest a developmental typicality in this area in children with DS, although they also note that the behaviors of the DS group were rated as significantly more intense than the behaviors of their TD counterparts.

The landscape of differences in the severity of ASD symptoms between DS and AD groups in the study is unclear. Even though children with DS scored lower in BISCUIT–Part 1 total scores compared to the AD group, the analysis of individual subscale scores showed no significant differences between the groups. Earlier findings revealed a characteristic pattern of strengths and weaknesses in adaptive functioning of toddlers with DS: stronger social skills, weaker expressive language, and poor motor coordination [54]. In addition, higher socialization scores differentiated the DS group from the mixed developmental disabilities group. There was no such clear pattern in our study, but the results should be interpreted with caution due to methodological limitations. The AD group was highly heterogeneous and included children with severe delay in intellectual and motor development, but also children with less severe developmental problems, mostly delayed speech. Future studies should enroll a more homogeneous AD group and match it to DS group for mental age.

Q-CHAT scores have also confirmed lower ASD symptom severity in the DS group than in the ASD group and higher compared to the TD group. The difference between DS and AD groups was not significant. There was a strong correlation between BISCUIT–Part 1 total score and Q-CHAT score (*r* = 0.788). Also, the correlations of BISCUIT–Part 2 and BISCUIT–Part 3 and Q-CHAT score were high or moderate. Matson et al. [51] reported that BISCUIT–Part 1 had good convergent validity with another screening tool for ASD, the Modified Checklist for Autism in Toddlers (M-CHAT; [55]) and the Personal-Social domain of the BDI-2, and a divergent validity with the adaptive and motor domains of the BDI-2. Our findings supplement those results with data on Q-CHAT.

An interesting pattern of results was obtained with respect to comorbid mental health conditions measured in BISCUIT–Part 2. Across all domains, the DS group scored lower than the ASD group. By contrast, we found no statistically significant differences in total score or any of the subscales between DS and TD groups. As for the differences between toddlers with DS and toddlers with ASD, data confirmed our expectations. Although percentages given for the prevalence of comorbidity of psychopathology in ASD vary significantly between studies (from 11% to 84% according to Davis et al., [56]), most authors report high rates of behavioral problems and psychiatric conditions in individuals with ASD, e.g., [57,58]. The most common problems include aggression and self-injurious behavior, obsessive-compulsive disorder, attention-deficit/hyperactivity disorder, tics, depression, and anxiety, as well as eating and sleep disorders. These conditions were therefore incorporated into BISCUIT–Part 2 due to their high incidence in the ASD population. The prevalence of comorbid psychopathologies in toddlers with ASD is also higher than in the general population and other clinical subgroups [56]. The rates of maladaptive behaviors reported for children with DS range between 18 and 43% [59]. There is very little empirical information comparing young children with ASD and DS. As was previously mentioned, in the research by Matson’s group [57,60,61] only a small percentage of the atypical development group were children with DS. Evans et al. [62] compared school-aged children with ASD, DS and TD, matched for developmental age and found higher anxiety levels in children with ASD. Our study provides new data about young children in these populations, demonstrating significantly higher severity of symptoms of all mental health conditions of interest in toddlers with ASD and no differences between DS and TD groups.

Dykens [35] reported that children with DS showed fewer behavior problems and comorbid psychopathologies than other groups with intellectual disabilities. This was also the case in our study: the DS group scored lower than AD group in BISCUIT–Part 2 both in total score and tantrum/conduct behavior. There were no differences between the two groups in the remaining subscales. Again, the differences in BISCUIT–Part 2 should be interpreted with caution due to the high heterogeneity of the AD group. It should also be noted that the prevalence of comorbid mental health conditions differs in individuals with DS depending on age [37]. In the case of young children participating in our study, the symptoms may be less noticeable for parents than in older children. This fact could explain the lack of differences between DS and TD groups in our study since other researchers have found higher severity of mental problems in children with DS than in typically developing peers [63].

A similar configuration of group differences emerged with respect to challenging behavior measured in BISCUIT–Part 3. In total score, as well as across all measured types of behavior (aggressive/disruptive, stereotypic and self-injurious), children with DS scored lower than children with ASD. These differences were consistent with our expectations. Behavioral problems, especially aggressive and stereotyped behaviors, are common in children with ASD, including young children with this disorder [64,65,66,67]. Children with DS are also at high risk of challenging behaviors [68], but these problems are less pronounced in them than in the ASD population. In the study sample of children with DS, parents noticed challenging behaviors relatively rarely; the group did not differ from TD children in that respect. Perhaps the children’s young age was a factor. In addition, the study was not controlled for mental age, and the sample may have included a large number of children with a slightly higher level of functioning.

The overall picture of our results with the DS group scoring higher in social and communication skills and manifesting less behavioral and emotional problems than the ASD group confirms the key adaptive role of these abilities, e.g., [69,70]. However, the level of socio-communication skills and the intensity of challenging behavior in the DS group varied greatly. It should be considered when planning support for toddlers with this syndrome. A detailed analysis of their social competencies can help explain the mechanisms of emotional and behavioral difficulties experienced by some children.

Nevertheless, the study was not free of limitations. The most prominent one is the lack of control for mental age and no matching of groups for that factor. The study population was also not controlled for physical health, including conditions such as epilepsy and other neurological and somatic disorders. All of the information were supplied by parents and no other measures of developmental difficulties typical for ASD or other mental conditions were used. ASD diagnosis was not verified using other standardized measures, and children with DS demonstrating high severity of ASD symptoms were not diagnosed for ASD. Assessments of comorbid mental health conditions and challenging behaviors used only one questionnaire each. Finally, the study was cross-sectional. The study would have offered more detailed information if we had tracked changes in ASD symptoms, symptoms of comorbid mental health problems and challenging behaviors over time, and collected information from other informants, e.g., teachers.

## 5. Conclusions

The main strength of this study is the young age of participants combined with its narrow range. The vast majority of studies in this area enroll older children, and the DS group is often highly varied in terms of age. We found lower severity of the symptoms of ASD as well as symptoms other mental health conditions and challenging behaviors in toddlers with DS than in toddlers with ASD. An additional outcome was to confirm the validity of BISCUIT–Parts 1–3, instruments for early screening for ASD symptoms and symptoms of comorbid mental health conditions in children with ASD. Our findings suggest that ASD symptoms are more severe in children with DS than their typically developing peers, especially with respect to socialization/nonverbal communication and verbal communication. As for comorbid mental health conditions and challenging behavior, we found no statistically significant differences between children with DS and TD children.

## Figures and Tables

**Table 1:** Characteristics of the sample.

	DS	ASD	AD	TD
N (males)	56 (33)	220 (168)	93 (57)	209 (108)
M age in months (*SD*), range	27.11 (5.792), 17–37	27.45 (5.690), 17–37	25.88 (4.982), 17–37	25.474 (5.713), 17–36
M birthweight [g] (*SD*), range	2946 (523) 1350–4230	3244 (637), 999–4600	2961 (867), 700–4450	3449 (511), 2090–4820
N preterm	0	25	9	0
N ASD familial risk	2	61	9	15
M mother’s age [years] (*SD*)	35 (0.5)	31 (0.5)	Data missing	Data missing
M father’s age [years] (*SD*)	36 (0.5)	32 (0.6)	Data missing	Data missing
Only child	29%	45%	39%	45%
Additional information	-	Hearing problems *n* = 5; vision problems: *n* = 2	Cerebral palsy *n* = 1; seizure disorder *n* = 1, vision problems *n* = 1	-

Abbreviations: DS, Down syndrome; ASD, autism spectrum disorders; AD, atypical development; TD, typical development; M—mean, *SD*—standard deviation.

**Table 2:** Correlations between participants’ age in months, BISCUIT and Q-CHAT scores.

		Age	BISCUIT—Part 1	BISCUIT—Part 2	BISCUIT—Part 3
BISCUIT–Part 1	*r*	0.012			
	*p*	0.831			
	n	341			
BISCUIT–Part 2	*r*	0.147 **	0.800 **		
	*p*	0.001	<0.001		
	n	578	341		
BISCUIT–Part 3	*r*	0.156 **	0.614 **	0.801 **	
	*p*	<0.001	<0.001	<0.001	
	n	576	340	576	
Q-CHAT	*r*	−0.123 *	0.788 **	0.687 *	0.577 *
	*p*	0.015	<0.001	<0.001	<0.001
	n	393	160	336	392

* *p* < 0.05; ** *p* < 0.001.

## Data Availability

Data are available from authors upon request.

## Appendix A

**Table A1:** Descriptive statistics for BISCUIT and Q-CHAT scores.

	DS			ASD			AD			TD		
	*M*	*SD*	Min–Max	*M*	*SD*	Min–Max	*M*	*SD*	Min–Max	*M*	*SD*	Min–Max
BISCUIT–Part 1 Total score	18.556	11.126	5–67	69.330	21.782	10–118	24.568	20.568	1–81	2.149	3.695	0–15
Socialization/Nonverbal communication	8.222	6.324	2–36	32.361	10.371	1–48	11.184	11.862	0–42	0.5	1.353	0–6
Repetitive behavior/Restricted interests	2.259	2.586	0–13	20.221	8.229	4–46	4.895	5.913	0–23	0.792	1.54	0–6
Verbal communication	6.074	2.386	1–13	10.274	3.299	0–14	5.79	3.772	0–12	0.6	1.364	0–6
Q-CHAT	28.024	7.413	13–42	43.500	12.685	17–80	29.262	11.032	5–62	23.641	8.940	5–60
BISCUIT–Part 2 Total score	8.556	7.350	0–37	32.748	17.294	3–104	13.434	13.686	0–70	6.444	8.019	0–43
Tantrum/conduct behavior	2.315	2.394	0–9	9.287	5.739	0–34	4.434	5.300	0–30	2.640	3.563	0–19
Inattention/impulsivity	3.574	3.178	0–12	11.762	6.412	0–30	4.816	5.447	0–27	2.028	3.287	0–19
Avoidance behavior	0.778	1.327	0–6	4.178	4.139	0–18	1.724	2.580	0–12	0.893	1.662	0–9
Anxiety/repetitive behavior	0.648	1.231	0–7	5.342	3.950	0–21	1.263	2.435	0–16	0.315	0.657	0–4
Eating/sleep problems	0.593	1.281	0–6	1.574	1.939	0–8	0.842	1.600	0–7	0.433	0.938	0–5
BISCUIT–Part 3 Total score	1.500	1.537	0–7	6.137	4.688	0–27	1.828	2.657	0–12	1.072	1.847	0–10
Aggressive/disruptive behaviors	1.250	1.225	0–5	3.562	3.452	0–19	1.419	2.108	0–11	0.971	1.725	0–9
Stereotypic behaviors	0.179	0.431	0–2	2.151	1.600	0–6	0.226	.662	0–4	0.063	0.280	0–2
Self-injurious behaviors	0.071	0.260	0–1	0.425	0.722	0–3	0.183	0.625	0–4	0.039	0.193	0–1

Abbreviations: DS, Down syndrome; ASD, autism spectrum disorders; AD, atypical development; TD, typical development; *M*—mean, *SD*—standard deviation.

## References

[1] American Psychiatric Association (2013). Diagnostic and Statistical Manual of Mental Disorders.

[2] World Health Organization (2018). International Classification of Diseases.

[3] Lai M.-C., Lombardo M.V., Baron-Cohen S. (2014). Autism. Lancet.

[4] Hazen E.P., Stornelli J.L., O’Rourke J.A., Koesterer K., McDougle C.J. (2014). Sensory Symptoms in Autism Spectrum Disorders. Harv. Rev. Psychiatry.

[5] Catalá-López F., Ridao M., Hurtado I., Núñez-Beltrán A., Gènova-Maleras R., Alonso-Arroyo A., Tobías A., Aleixandre-Benavent R., Catalá M.A., Tabarés-Seisdedos R. (2019). Prevalence and comorbidity of autism spectrum disorder in Spain: Study protocol for a systematic review and meta-analysis of observational studies. Syst. Rev..

[6] Maenner M.J., Shaw K.A., Baio J., Washington A., Patrick M., DiRienzo M., Christensen D.L., Wiggins L.D., Pettygrove S., Andrews J.G. (2020). Prevalence of Autism Spectrum Disorder Among Children Aged 8 Years—Autism and Developmental Disabilities Monitoring Network, 11 Sites, United States, 2016. MMWR. Surveill. Summ..

[7] Tordjman S., Cohen D., Coulon N., Anderson G.M., Botbol M., Canitano R., Roubertoux P.L. (2017). Reframing autism as a behavioral syndrome and not a specific mental disorder: Implications of genetic and phenotypic heterogeneity. Neurosci. Biobehav. Rev..

[8] Richards C., Jones C., Groves L., Moss J., Oliver C. (2015). Prevalence of autism spectrum disorder phenomenology in genetic disorders: A systematic review and meta-analysis. Lancet Psychiatry.

[9] Mégarbané A., Ravel A., Mircher C., Sturtz F., Grattau Y., Rethoré M.-O., Delabar J.-M., Mobley W.C. (2009). The 50th anniversary of the discovery of trisomy 21: The past, present, and future of research and treatment of Down syndrome. Genet. Med..

[10] Bull M.J. (2020). Down Syndrome. N. Engl. J. Med..

[11] Chapman R.S., Hesketh L.J. (2000). Behavioral phenotype of individuals with Down syndrome. Ment. Retard. Dev. Disabil. Res. Rev..

[12] Silverman W. (2007). Down syndrome: Cognitive phenotype. Ment. Retard. Dev. Disabil. Res. Rev..

[13] Karmiloff-Smith A., al-Janabi T., D’Souza H., Groet J., Massand E., Mok K., Startin C., Fisher E., Hardy J., Nizetic D. (2016). The importance of understanding individual differences in Down syndrome. F1000Research.

[14] DiGuiseppi C., Hepburn S., Davis J.M., Fidler D.J., Hartway S., Lee N., Miller L., Ruttenber M., Robinson C. (2010). Screening for Autism Spectrum Disorders in Children with Down Syndrome. J. Dev. Behav. Pediatr..

[15] Kent L., Evans J., Paul M., Sharp M. (2007). Comorbidity of autistic spectrum disorders in children with Down syndrome. Dev. Med. Child Neurol..

[16] Oxelgren U.W., Myrelid Å., Annerén G., Ekstam B., Göransson C., Holmbom A., Isaksson A., Åberg M., Gustafsson J., Fernell E. (2016). Prevalence of autism and attention-deficit-hyperactivity disorder in Down syndrome: A population-based study. Dev. Med. Child Neurol..

[17] Warner G., Moss J., Smith P., Howlin P. (2014). Autism Characteristics and Behavioural Disturbances in ∼ 500 Children with Down’s Syndrome in England and Wales. Autism Res..

[18] Howlin P., Wing L., Gould J. (2008). The recognition of autism in children with down syndrome-implications for intervention and some speculations about pathology. Dev. Med. Child Neurol..

[19] Mammad K., Chkirat M., Kriouile Y., Alaoui A.M. (2019). Children with Down Syndrome (DS), and Autism Spectrum Disorder (ASD): Difficulties of Screening and Management of This Dual Diagnosis about 3 Cases. Psychology.

[20] Lord C., Rutter M., DiLavore P., Risi S., Gotham K., Bishop S. (2012). Autism Diagnostic Observation Schedule.

[21] Oxelgren U.W., Åberg M., Myrelid Å., Annerén G., Westerlund J., Gustafsson J., Fernell E. (2019). Autism needs to be considered in children with Down Syndrome. Acta Paediatr..

[22] Godfrey M., Hepburn S., Fidler D.J., Tapera T., Zhang F., Rosenberg C.R., Lee N.R. (2019). Autism spectrum disorder (ASD) symptom profiles of children with comorbid Down syndrome (DS) and ASD: A comparison with children with DS-only and ASD-only. Res. Dev. Disabil..

[23] Hamner T., Hepburn S., Zhang F., Fidler D., Rosenberg R., Robins D.L., Lee N.R. (2020). Cognitive Profiles and Autism Symptoms in Comorbid Down Syndrome and Autism Spectrum Disorder. J. Dev. Behav. Pediatr..

[24] Magyar C.I., Pandolfi V., Dill C.A. (2012). An Initial Evaluation of the Social Communication Questionnaire for the Assessment of Autism Spectrum Disorders in Children with Down Syndrome. J. Dev. Behav. Pediatr..

[25] Molloy C.A., Murray D.S., Kinsman A., Castillo H., Mitchell T., Hickey F.J., Patterson B. (2009). Differences in the clinical presentation of Trisomy 21 with and without autism. J. Intellect. Disabil. Res..

[26] Warner G., Howlin P., Salomone E., Moss J., Charman T. (2016). Profiles of children with Down syndrome who meet screening criteria for autism spectrum disorder (ASD): A comparison with children diagnosed with ASD attending specialist schools. J. Intellect. Disabil. Res..

[27] Channell M.M., Phillips B.A., Loveall S.J., Conners F.A., Bussanich P.M., Klinger L.G. (2015). Patterns of autism spectrum symptomatology in individuals with Down syndrome without comorbid autism spectrum disorder. J. Neurodev. Disord..

[28] Constantino J., Gruber C. (2005). Social Responsiveness Scale.

[29] Channell M.M. (2020). The Social Responsiveness Scale (SRS-2) in school-age children with Down syndrome at low risk for autism spectrum disorder. Autism Dev. Lang. Impair..

[30] Constantino J.N., Gruber C.P. (2012). Social Responsiveness Scale.

[31] Hepburn S., Philofsky A., Fidler D.J., Rogers S. (2007). Autism symptoms in toddlers with Down syndrome: A descriptive study. J. Appl. Res. Intellect. Disabil..

[32] Lord C., Risi S., Lambrecht L., Cook E.H., Leventhal B.L., DiLavore P.C., Pickles A., Rutter M. (2000). The Autism Diagnostic Observation Schedule—Generic: A Standard Measure of Social and Communication Deficits Associated with the Spectrum of Autism. J. Autism Dev. Disord..

[33] Lord C., Rutter M., Le Couteur A. (1994). Autism Diagnostic Interview-Revised: A revised version of a diagnostic interview for caregivers of individuals with possible pervasive developmental disorders. J. Autism Dev. Disord..

[34] Matson J.L., Wilkins J., Sevin J.A., Knight C., Boisjoli J.A., Sharp B. (2009). Reliability and item content of the Baby and Infant Screen for Children with aUtIsm Traits (BISCUIT): Parts 1–3. Res. Autism Spectr. Disord..

[35] Dykens E.M. (2007). Psychiatric and behavioral disorders in persons with Down syndrome. Ment. Retard. Dev. Disabil. Res. Rev..

[36] Bhatia M.S., Kabra M., Sapra S. (2005). Behavioral problems in children with Down syndrome. Indian Pediatr..

[37] Startin C.M., D’Souza H., Ball G., Hamburg S., Hithersay R., Hughes K.M.O., Massand E., Karmiloff-Smith A., Thomas M.S.C., Consortium L. (2020). Health comorbidities and cognitive abilities across the lifespan in Down syndrome. J. Neurodev. Disord..

[38] Achenbach T.M. (1991). Manual for the Child Behavior Checklist/4-18 and 1991 Profile.

[39] Dieleman L.M., De Pauw S.S., Soenens B., Van Hove G., Prinzie P. (2018). Behavioral Problems and Psychosocial Strengths: Unique Factors Contributing to the Behavioral Profile of Youth with Down Syndrome. Am. J. Intellect. Dev. Disabil..

[40] Chebli S.S., Martin V., Lanovaz M.J. (2016). Prevalence of Stereotypy in Individuals with Developmental Disabilities: A Systematic Review. Rev. J. Autism Dev. Disord..

[41] Capone G.T., Grados M.A., Kaufmann W.E., Bernad-Ripoll S., Jewell A. (2005). Down syndrome and comorbid autism-spectrum disorder: Characterization using the aberrant behavior checklist. Am. J. Med Genet. Part A.

[42] Carter J.C., Capone G.T., Gray R.M., Cox C.S., Kaufmann W.E. (2007). Autistic-spectrum disorders in down syndrome: Further delineation and distinction from other behavioral abnormalities. Am. J. Med. Genet. Part B Neuropsychiatr. Genet..

[43] Allison C., Baron-Cohen S., Wheelwright S., Charman T., Richler J., Pasco G., Brayne C. (2008). The Q-CHAT (Quantitative CHecklist for Autism in Toddlers): A Normally Distributed Quantitative Measure of Autistic Traits at 18–24 Months of Age: Preliminary Report. J. Autism Dev. Disord..

[44] World Health Organization (1992). International Classification of Diseases: Diagnostic Criteria for Research.

[45] Matson J.L., Wilkins J., Sharp B., Knight C., Sevin J.A., Boisjoli J.A. (2009). Sensitivity and specificity of the Baby and Infant Screen for Children with aUtIsm Traits (BISCUIT): Validity and cutoff scores for autism and PDD-NOS in toddlers. Res. Autism Spectr. Disord..

[46] Matson J.L., Wilkins J., Fodstad J.C. (2011). The Validity of the Baby and Infant Screen for Children with aUtIsm Traits: Part 1 (BISCUIT: Part 1). J. Autism Dev. Disord..

[47] Matson J.L., Boisjoli J.A., Hess J.A., Wilkins J. (2010). Factor structure and diagnostic fidelity of the Baby and Infant Screen for Children with aUtIsm Traits–Part 1 (BISCUIT–part 1). Dev. Neurorehabilit..

[48] Matson J.L., Fodstad J.C., Mahan S. (2009). Cutoffs, norms, and patterns of comorbid difficulties in children with developmental disabilities on the Baby and Infant Screen for Children with aUtIsm Traits (BISCUIT-Part 2). Res. Dev. Disabil..

[49] Rojahn J., Matson J.L., Mahan S., Fodstad J.C., Knight C., Sevin J.A., Sharp B. (2009). Cutoffs, norms, and patterns of problem behaviors in children with an ASD on the Baby and Infant Screen for Children with aUtIsm Traits (BISCUIT-Part 3). Res. Autism Spectr. Disord..

[50] Brislin R.W. (1970). Back-Translation for Cross-Cultural Research. J. Cross-Cult. Psychol..

[51] Matson J.L., Boisjoli J., Wilkins J. (2007). The Baby and Infant Screen for Children with aUtIsm Traits (BISCUIT).

[52] Glenn J. (2017). Repetitive Behaviours and Restricted Interests in Individuals with Down Syndrome—One Way of Managing Their World?. Brain Sci..

[53] Evans D.W., Gray F.L. (2000). Compulsive-like behavior in individuals with Down syndrome: Its relation to mental age level, adaptive and maladaptive behavior. Child Dev..

[54] Fidler D.J., Hepburn S., Rogers S. (2006). Early learning and adaptive behaviour in toddlers with Down syndrome: Evidence for an emerging behavioural phenotype?. Down Syndr. Res. Pract..

[55] Robins D.L., Fein D., Barton M.L., Green J.A. (2001). The Modified Checklist for Autism in Toddlers: An Initial Study Investigating the Early Detection of Autism and Pervasive Developmental Disorders. J. Autism Dev. Disord..

[56] Davis T.E., Fodstad J.C., Jenkins W.S., Hess J.A., Moree B.N., Dempsey T., Matson J.L. (2010). Anxiety and avoidance in infants and toddlers with autism spectrum disorders: Evidence for differing symptom severity and presentation. Res. Autism Spectr. Disord..

[57] Belardinelli C., Raza M., Taneli T. (2016). Comorbid Behavioral Problems and Psychiatric Disorders in Autism Spectrum Disorders. J. Child. Dev. Disord..

[58] Casanova M.F., Frye R.E., Gillberg C., Casanova E.L. (2020). Editorial: Comorbidity and Autism Spectrum Disorder. Front. Psychiatry.

[59] Esbensen A.J., Hoffman E.K., Shaffer R., Chen E., Patel L., Jacola L. (2018). Reliability of parent report measures of behaviour in children with Down syndrome. J. Intellect. Disabil. Res..

[60] Matson J.L., Boisjoli J., Rojahn J., Hess J. (2009). A factor analysis of challenging behaviors assessed with the Baby and Infant Screen for Children with aUtism Traits (BISCUIT-Part 3). Res. Autism Spectr. Disord..

[61] Matson J.L., Matheis M., Burns C.O., Esposito G., Venuti P., Pisula E., Misiak A., Kalyva E., Tsakiris V., Kamio Y. (2017). Examining cross-cultural differences in autism spectrum disorder: A multinational comparison from Greece, Italy, Japan, Poland, and the United States. Eur. Psychiatry.

[62] Evans D.W., Canavera K., Kleinpeter F.L., Maccubbin E., Taga K. (2005). The Fears, Phobias and Anxieties of Children with Autism Spectrum Disorders and Down Syndrome: Comparisons with Developmentally and Chronologically Age Matched Children. Child Psychiatry Hum. Dev..

[63] Fodstad J.C., Rojahn J., Matson J.L. (2010). Emergent Comorbidity in At Risk Children with and without Autism Spectrum Disorder—A Cross-Sectional Study. J. Dev. Phys. Disabil..

[64] Matson J.L., Hess J.A., Boisjoli J.A. (2010). Comorbid psychopathology in infants and toddlers with autism and pervasive developmental disorders-not otherwise specified (PDD-NOS). Res. Autism Spectr. Disord..

[65] Chandler S., Howlin P., Simonoff E., O’Sullivan T., Tseng E., Kennedy J., Charman T., Baird G. (2015). Emotional and behavioural problems in young children with autism spectrum disorder. Dev. Med. Child Neurol..

[66] Hartley S.L., Sikora D.M., McCoy R. (2008). Prevalence and risk factors of maladaptive behaviour in young children with Autistic Disorder. J. Intellect. Disabil. Res..

[67] Hill A.P., Zuckerman K.E., Hagen A.D., Kriz D.J., Duvall S.W., van Santen J., Nigg J., Fair D., Fombonne E. (2014). Aggressive behavior problems in children with autism spectrum disorders: Prevalence and correlates in a large clinical sample. Res. Autism Spectr. Disord..

[68] Feeley K., Jones E. (2008). Strategies to address challenging behaviour in young children with Down syndrome. Down Syndr. Res. Pract..

[69] Montagna A., Nosarti C. (2016). Socio-Emotional Development Following Very Preterm Birth: Pathways to Psychopathology. Front. Psychol..

[70] Rodas N.V., Eisenhower A., Blacher J. (2017). Structural and Pragmatic Language in Children with ASD: Longitudinal Impact on Anxiety and Externalizing Behaviors. J. Autism Dev. Disord..

